# CTLA-4 Modulates the Differentiation of Inducible Foxp3^+^ Treg Cells but IL-10 Mediates Their Function in Experimental Autoimmune Encephalomyelitis

**DOI:** 10.1371/journal.pone.0108023

**Published:** 2014-09-19

**Authors:** Johan Verhagen, Leona Gabryšová, Ella R. Shepard, David C. Wraith

**Affiliations:** School of Cellular and Molecular Medicine, University of Bristol, Bristol, United Kingdom; New York University, United States of America

## Abstract

In vitro induced Foxp3^+^ T regulatory (iTreg) cells form a novel and promising target for therapeutic tolerance induction. However, the potential of these cells as a target for the treatment of various immune diseases, as well as the factors involved in their development and function, remain debated. Here, we demonstrate in a myelin basic protein (MBP)-specific murine model of CNS autoimmune disease that adoptive transfer of antigen-specific iTreg cells ameliorates disease progression. Moreover, we show that the co-stimulatory molecule CTLA-4 mediates in vitro differentiation of iTreg cells. Finally, we demonstrate that the secreted, immunosuppressive cytokine IL-10 controls the ability of antigen-specific iTreg cells to suppress autoimmune disease. Overall, we conclude that antigen-specific iTreg cells, which depend on various immune regulatory molecules for their differentiation and function, represent a major target for effective immunotherapy of autoimmune disease.

## Introduction

Thymic deletion of self-antigen-specific CD4^+^ T cells and the generation of Foxp3^+^ thymic T regulatory (tTreg) cells are instrumental in the prevention of undesirable immune responses to self antigens. Previous work in the Tg4 TCR-transgenic mouse model, specific for the myelin basic protein (MBP)-derived peptide Ac1–9, demonstrated that augmented differentiation of tTreg cells provides greater resistance to the induction of experimental autoimmune encephalomyelitis (EAE), the murine model of multiple sclerosis (MS) [1]. Extra-thymic induction of a regulatory phenotype in peripheral conventional T (Tconv) cells acts as a second tier of defense against undesirable immune responses to host tissues. Two main subsets of extra-thymic CD4^+^ T regulatory cells have now been described; Interleukin-10 (IL-10)-secreting, Foxp3^−^ Treg cells and extra-thymic Foxp3^+^ Treg cells. The latter are commonly divided into in vivo-generated peripheral Treg (pTreg) cells and in vitro-induced Treg (iTreg) cells [2]. Although iTreg cells and pTreg cells are both Foxp3^+^ cells differentiated from naive conventional T cells, they may not be comparable functionally. Due to their inducible nature, extra-thymic Treg cells form a prime target for immunotherapeutic tolerance induction in cases where central tolerance has proven insufficient. We have demonstrated previously that IL-10-secreting CD4^+^ T cells of Th1 origin can be generated in vivo by repeated administration of a high MHC-affinity variant of the MBP Ac1–9 peptide and that these cells protect mice against the development of EAE [3]. In this study, we explore if adoptive transfer of Foxp3^+^ iTreg cells, specific for the same antigen and generated in vitro by stimulation of naive CD4^+^ Tconv cells in the presence of IL-2 and TGF-β_1_, can equally offer protection from CNS autoimmune disease.

Although iTreg cells have been the subject of intense investigation for several years, many aspects regarding their development and function remain unknown or debated. A better understanding of these issues is of pivotal importance for the future of iTreg cell-based therapy. In the thymus, development of Foxp3^+^ cells depends not only on the expression of specific self-antigen but also on co-factors including cytokines, adhesion molecules and co-stimulation. Recently, we demonstrated a crucial role for the negative co-stimulatory molecule CTLA-4 in setting the threshold for thymic selection of both Foxp3^+^ Treg cells and CD4^+^ Tconv cells [4]. CTLA-4 has previously been suggested to be indispensible for TGF-β-mediated Foxp3 induction in peripheral Tconv cells in vitro [5], although this finding was contradicted recently [6]. The latter group further demonstrated that CTLA-4 is important for iTreg cell-mediated downregulation of dendritic cell function in vitro, although less so than IL-10. This mechanism is reminiscent of that demonstrated previously for IL-10-secreting Foxp3^−^ T cells [3]. Like CTLA-4, IL-10 has previously been suggested to be important not only as a mediator of suppression for Foxp3^+^ iTreg cells [7], [8] but also to enhance stable Foxp3 expression in CD4^+^ T cells in a murine model of colitis [9].

In this study, we demonstrate that antigen-specific iTreg cells can provide protection against EAE in our MBP-specific model. Moreover, we show that while CTLA-4, but not IL-10, plays a role in the induction of Foxp3 expression in naive CD4^+^ T cells, the latter is important for iTreg cell-mediated protection from autoimmune disease.

## Materials and Methods

### Ethics statement

All animal experiments were carried out under the UK Home Office Project Licence number 30/2705 held by D.C.W. and the study was approved by the University of Bristol ethical review committee.

### Mice

B10.PL, Tg4, Tg4 CD45.1, Tg4 Rag-1^−/−^, Tg4 IL-10^−/−^ and Tg4 CTLA-4^−/−^ mice were bred and kept under specific pathogen-free conditions at the University of Bristol Animal Services Unit.

### Peptide

The acetylated N-terminal peptide of murine MBP, Ac1–9 (Ac-ASQKRPSQR) was custom synthesized (GL Biochem (Shanghai) Ltd).

### Naive T cell isolation

Naive CD4^+^ T cells were isolated magnetically using a naive T cell isolation kit (Stemcell Technologies) according to the manufacturer's recommended protocol.

### iTreg cell differentiation

Naive splenic CD4^+^ T cells from mice 4–7 weeks of age were cultured in vitro for 7 days in the presence of 100 U/ml rhIL-2 (R&D systems) and 10 ng/ml rhTGF-β_1_ (Peprotech). Cells were stimulated with anti-CD3e (1 µg/ml) and anti-CD28 (2 µg/ml) plate-bound antibody (eBioscience or BioXcell). The level of Foxp3 induction was assessed by flow cytometry prior to in vitro co-culture or transfer in vivo and averaged 95% (range 92–98%) purity for transferred B10.PL, Tg4 and Tg4 IL-10^−/−^ iTreg cells and 85% (range 84–85%) for Tg4 CTLA-4^−/−^ iTreg cells

### Flow cytometry

Flow cytometric analysis was performed using an LSR II flow cytometer (BD). Cell phenotypes were analyzed using combinations of anti-GITR-PE or -efluor450, anti-PD-1-PE, anti-FoxP3-PE, -efluor450 or -APC, anti-CD8-PE, anti-C45.1 PECy7, anti-Helios-FITC, anti-Eos-PE, anti-CD103-APC, anti-CD4-AlexaFluor700 (all from eBioscience), and anti-Neuropilin-1-PE or -APC, anti-CD103-PerCPCy5.5 (Biolegend) antibodies. Fixable viability dye efluor780 (eBioscience) was used in all experiments to exclude dead cells. Cell proliferation dye (CPD)-efluor450 and CFSE (both from eBioscience) were used to monitor cell proliferation. Anti-Ki67-PE (eBioscience) was used to identify dividing cells. Results were analyzed using FlowJo analysis software (Tree Star, Inc.).

### iTreg cell transfer and EAE

Mice received an adoptive transfer of 5×10^6^ iTreg cells, i.p., 2–3 days prior to disease induction. Disease was induced by subcutaneous (s.c.) injection of 200 µg of MBP Ac1–9 in 0.1 ml of a sonicated emulsion consisting of an equal volume of complete Freund's adjuvant (CFA) and PBS containing 4 mg/ml of heat-killed Mycobacterium Tuberculosis (both from Difco) at the base of the tail. On days 0 and 2, 200 ng of Pertussis toxin (Sigma Aldrich) was administered intraperitoneally (i.p.) in 0.5 ml of PBS. EAE was assessed twice daily for up to 21 days using the following scoring system: 0, no signs; 1, flaccid tail; 2; impaired righting reflex and/or partial hind limb paralysis; 3, hind limb paralysis; 4, forelimb and hind limb paralysis; 5, moribund. Mice with disease grade 2 or above were given easy access to mashed-up food pellets and water. Mice with grade 4 disease for more than 48 hours or grade 5 disease were culled humanely by cervical dislocation. Due to the sometimes fragile phenotype of recipient and donor mice individual experiments were performed using small and sometimes varying group sizes of 2–5 individuals and pooled for final analysis. Statistical analysis was performed on the disease burden; cumulative disease score for each individual mouse over the duration of the experiment.

For the spontaneous disease-free survival experiments, Tg4 IL-10^−/−^ mice were checked daily for signs of EAE. In some cases, mice received an adoptive transfer of 1.5×10^6^ splenic CD4^+^CD25^+^ Treg cells isolated from the spleens of sex-matched, Tg4 IL-10^+/+^ donors using a Treg cell selection kit (Miltenyi Biotec) at the age of 4–5 weeks. Upon the first detection of disease, animals were culled humanely by cervical dislocation.

Histological analysis of brain and spinal cord from mice with spontaneous CNS autoimmune disease was assessed using two distinct scoring systems. Longitudinal sections of brain and spinal cord were stained with haematoxylin/eosin (inflammation score) or luxol fast blue/cresyl violet (demyelination score). Inflammation score grades: 0; no inflammatory cells, 1; a few scattered inflammatory cells, 2; organisation of inflammatory infiltrates around blood vessels into cuffs, 3; perivascular cuffs with infiltrate into parenchyma or parenchymal infiltrate without cuffing. Demyelination score grades: 0; no demyelination, 1; a few scattered naked axons, 2; small groups of naked axons, 3; large groups of naked axons, 4; confluent foci of demyelination.

### Statistical analysis

Data was analyzed for statistical significance using GraphPad Prism software with an appropriate test as indicated.

## Results and Discussion

### Antigen-specific iTreg cells delay the development of MBP-mediated EAE

**Figure 1:**
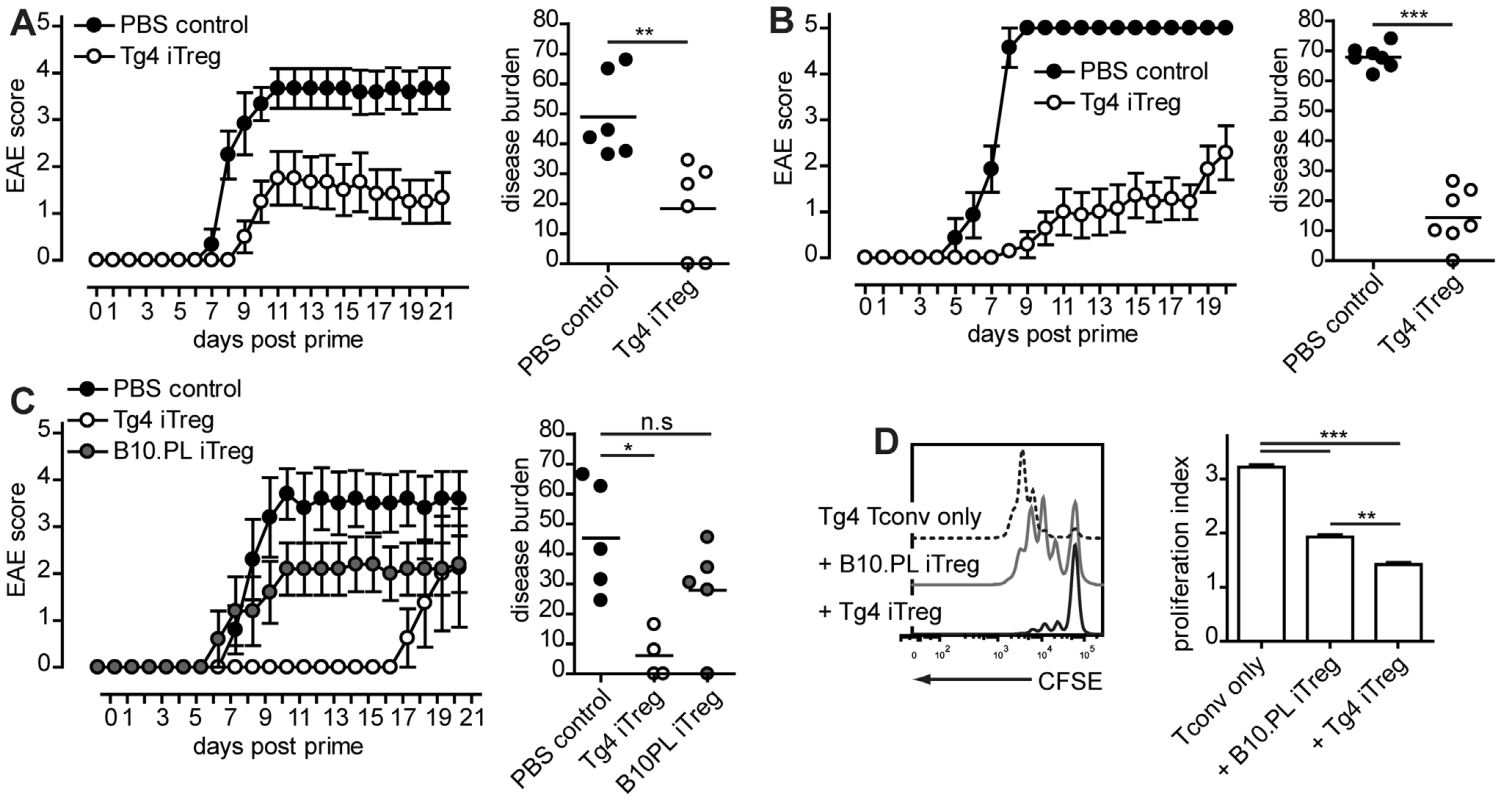
Antigen-specific iTreg cells ameliorate EAE progression. A–C. Mice received 5×10^6^ iTreg cells in PBS or PBS only i.p. 2–3 days prior to EAE induction with MBP Ac1–9 in CFA. A. Tg4 Rag^+/+^ recipients. N = 6 per group, combined from 2 individual experiments. ** P = 0.0022, Mann Whitney test. B. Tg4 Rag^−/−^ recipients. n = 7 per group, combined from 3 individual experiments. *** P = 0.0006, Mann Whitney test. C. Tg4 Rag^−/−^ recipients. PBS control and B10.PL iTreg n = 5, Tg4 iTreg n = 4, combined from 2 individual experiments. * P< 0.05, Kruskal Wallis with Dunn's post-test. A-C EAE plots depict mean ± SEM. Horizontal lines in disease burden scatter plots indicate mean. D. Tg4 CD45.1 naive CD4^+^ T cells, labeled with CFSE, were cultured alone or co-cultured at a 1 to 1 ratio with B10.PL or Tg4 (both CD45.2^+^) iTreg cells for 4 days and stimulated with 1 µg/ml MBP Ac1–9. CFSE dilution was measured by FACS (gated on CD45.1^+^ cells) and proliferation indexes calculated using FlowJo software. Bar graph shows mean ± SEM. One experiment of 3 similar experiments in triplicate is shown. ** P<0.01, *** P<0.001, Tukey's multiple comparison test.

Previous studies have demonstrated that adoptive transfer of antigen-specific or polyclonal iTreg cells can protect mice against the induction of EAE using the myelin sheath proteins proteolipid protein (PLP) or myelin oligodendrocyte glycoprotein (MOG) [7], [10]. The immunodominant peptides of these proteins, PLP 139–151 and MOG 35–55, are not expressed, or expressed at only very low levels, in the thymus of young mice in particular [11]–[13]. Therefore, T cells recognizing these peptides are not negatively selected, resulting in a high number of antigen-specific peripheral Tconv cells. It is further reasonable to assume that the lack of thymic expression results in low numbers of antigen-specific tTreg cells. In contrast, thymic expression of MBP ensures robust thymic generation of tTreg cells, although the very low affinity of the dominant Ac1–9 peptide does allow antigen-specific Tconv cells to escape deletion [1], [4]. In TCRαβ-transgenic Tg4 mice, in which 90–95% of CD4^+^ T cells recognize MBP Ac1–9, the generation of tTreg cells suffices to protect animals from the spontaneous CNS autoimmune disease observed in Tg4 Rag-1^−/−^ mice, which lack Foxp3 expression [1]. Recently, we showed that a modification of the Foxp3 protein that selectively impaired the generation of extra-thymic pTreg cells but not tTreg cells did not augment the susceptibility of these mice to spontaneous EAE [14]. On the other hand, previous work showed that the induction of IL-10-secreting T cells in this model increased resistance to induced disease [3]. In this study, we addressed the question whether adoptive transfer of iTreg cells in the MBP Ac1-9-specific Tg4 model can equally provide enhanced protection from induced EAE. To achieve this, iTreg cells were differentiated by stimulation of naive CD4^+^ splenic T cells in vitro with plate-bound anti-CD3 and anti-CD28 antibody in the presence of 100 U/ml rhIL-2 and 10 ng/ml rhTGF-β_1_ for 7 days. After culture, Foxp3 expression in T cells typically averaged approximately 95%. To assess the efficacy of these cells in controlling EAE development, 5×10^6^ Tg4 iTreg cells were transferred intraperitoneally to either Tg4 recipients, which experience thymic development of antigen-specific, Foxp3^+^ tTreg cells, or Tg4 Rag^−/−^ animals, which lack endogenous Foxp3 expression, 2–3 days prior to EAE induction with MBP Ac1–9 in CFA. In both scenarios, iTreg cells delayed disease progression (Figures 1A and 1B), demonstrating that iTreg cells mediate immune regulation independent of tTreg cells. In order to examine whether antigen-specificity plays a role in disease protection, Tg4 Rag^−/−^ mice, 5–8 weeks of age, received 5×10^6^ iTreg cells from either Tg4 or non-transgenic B10.PL mice 3 days prior to disease induction with MBP Ac1–9 in CFA. Whereas there appeared to be a minor benefit from the administration of polyclonal B10.PL iTreg cells, this only reached statistical significance when protection was offered by antigen-specific Tg4 iTreg cells (Figure 1C). This effect was further reflected in in vitro suppression assays (Figure 1D). These results are reminiscent of those achieved previously in a PLP-specific system [10], although polyclonal iTreg cells were reported effective in protection against induction of EAE using MOG 35–55 in a non-transgenic model [7].

### CTLA-4, but not IL-10, modulates iTreg cell differentiation

**Figure 2:**
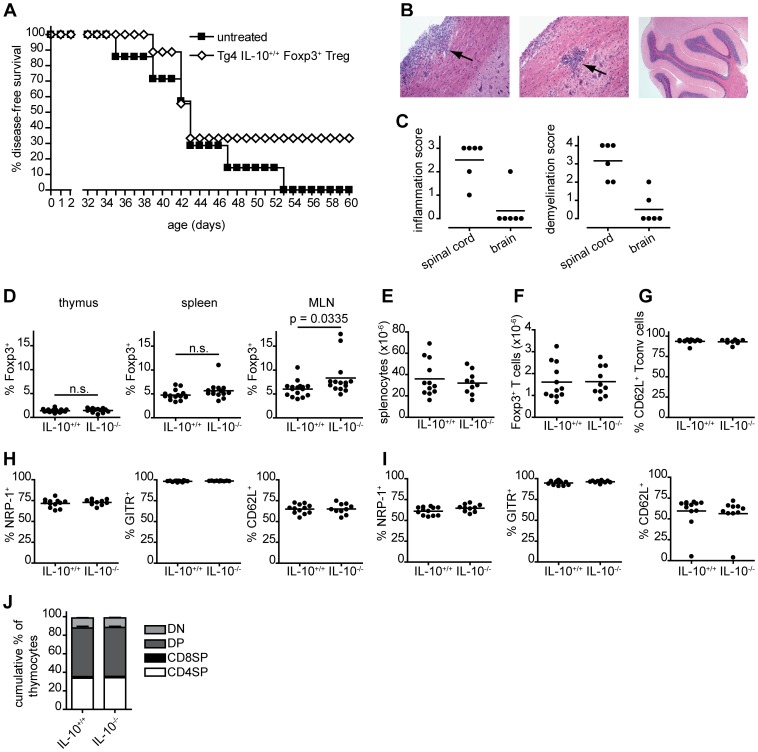
Tg4 IL-10^−/−^ mice exhibit spontaneous EAE but no deficiency in Foxp3^+^ Treg cell numbers or phenotype. A. Tg4 IL-10^−/−^ mice, untreated, n = 7, or receiving 1.5×10^6^ Tg4 IL-10^+/+^ splenic Treg cells i.p. at age 4–5 weeks, n = 9, were monitored daily for the spontaneous development of CNS autoimmune disease. Logrank test, P = 0.25 B. Representative haematoxylin/eosin stained sections of spinal cord (left and middle) and brain (right) from Tg4 IL-10^−/−^ with established CNS autoimmune disease (n = 6). Arrows indicate lymphocytic infiltrate. Magnifications: left; ×200, middle; ×100, right; ×25. C. Inflammation and demyelination scores of spinal cord and brain sections of Tg4 IL-10^−/−^ with established disease. Horizontal lines indicate means. n = 6 each. D-J. Tg4 IL-10^+/+^ and Tg4 IL-10^−/−^ mice approximately 5 weeks of age were examined for a number of characteristics; D. Frequency of Foxp3^+^ cells among CD4^+^CD8^−^ (CD4SP) thymocytes or CD4^+^ T cells in the spleen and mesenteric lymph nodes (MLN). Thymus; Tg4 IL-10^+/+^ n = 16, Tg4 IL-10^−/−^ n = 13. Spleen and MLN; Tg4 IL-10^+/+^ n = 16, Tg4 IL-10^−/−^ n = 14. E. Total number of splenocytes. F. Number of CD4^+^Foxp3^+^ cells in the spleen. G. Percentage CD62L expression on splenic CD4^+^ Foxp3^−^ cells. H. Percentage Neuropilin-1 (NRP-1), GITR and CD62L expression on splenic CD4^+^Foxp3^+^ cells. I. Percentage NRP-1, GITR and CD62L expression on CD4^+^Foxp3^+^ cells in the MLN. J. Cumulative frequency of CD4 and CD8 single positive (SP), double positive (DP) or double negative (DN) thymocytes. E–J. Tg4 IL-10^+/+^ n = 12, Tg4 IL-10^−/−^ n = 10. D–I. statistical analysis by unpaired, 2-tailed, t-test. Panels E-I; no significant differences.

The data above demonstrate that adoptive transfer of antigen-specific iTreg cells can ameliorate the progression of CNS autoimmune disease. In the next step, we sought to define factors, other than TCR signals, that determine iTreg cell differentiation. Both the negative co-stimulatory molecule CTLA-4 [15] and the suppressive cytokine IL-10 [16] have previously been shown to play a central role in immune regulation. In the Tg4 model, CTLA-4 sets the threshold for thymic development of tTreg cells and Tconv cells and thereby controls susceptibility to autoimmune disease [1], [4]. Despite deficiency of CTLA-4, Tg4 CTLA-4^−/−^ mice do not develop autoimmune disease due to enhanced selection of tTreg cells [1]. This is in contrast to Tg4 IL-10^−/−^ mice that develop spontaneous CNS autoimmune disease at the early age of approximately 5–7 weeks (Figure 2A – 2C). Unlike CTLA-4, IL-10 does not appear to have an effect on thymic development of tTreg cells and Tconv cells as thymic composition, spleen size, Treg cell number and Treg cell phenotype are normal for as far as we have been able to determine (Figure 2D–2J). Still, the adoptive transfer of splenic CD4^+^CD25^+^Foxp3^+^ Tg4 IL-10^+/+^ Treg cells appeared to limit disease progression in Tg4 IL-10^−/−^ mice, although this did not reach statistical significance in our experimental set-up (Figure 2A). The lack of complete protection could suggest that IL-10 from cell sources other than thymic CD4^+^ Treg cells, including extra-thymically differentiated Treg cell subsets, is important for the control of immune responses to self antigens.

**Figure 3:**
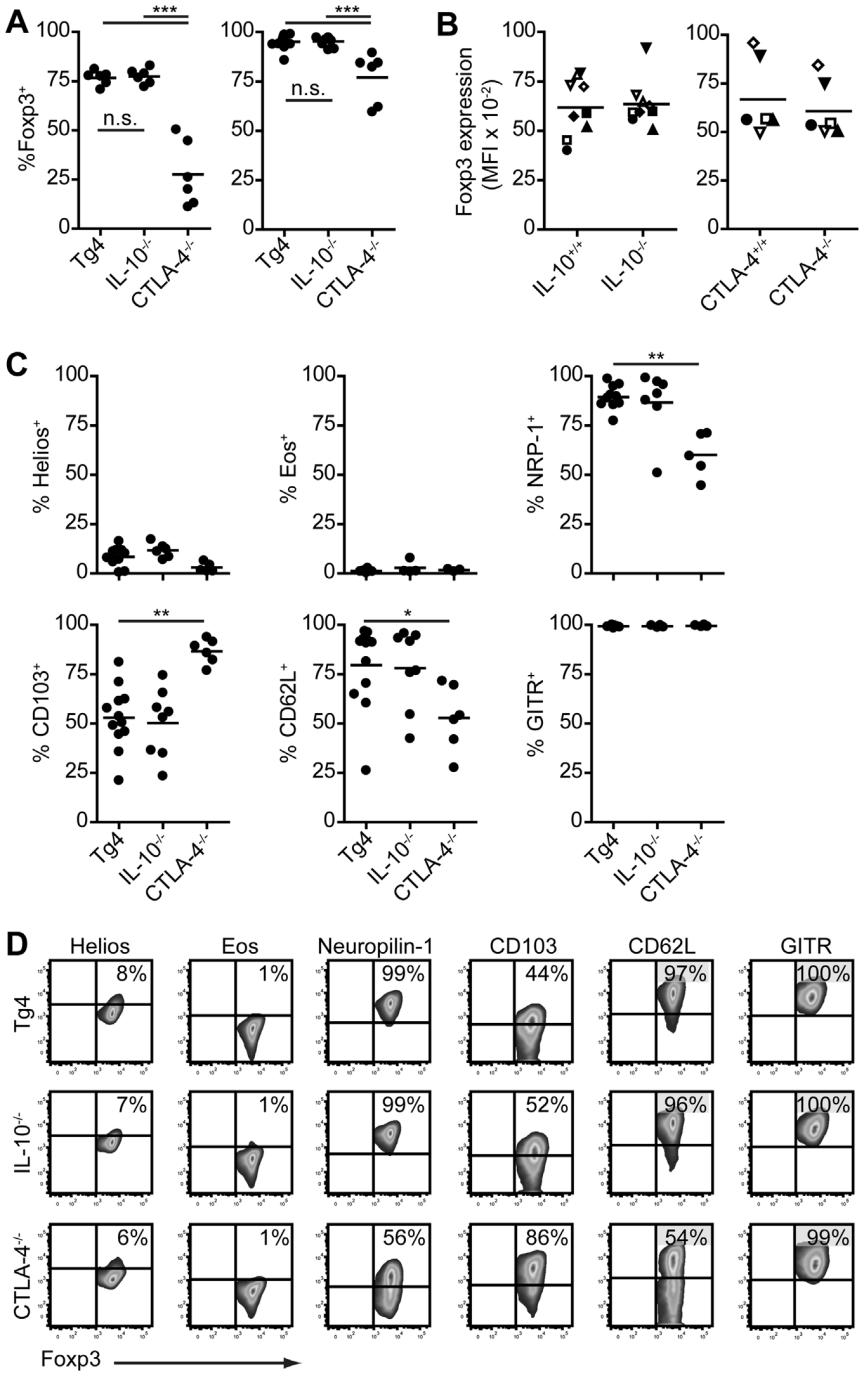
CTLA-4 but not IL-10 deficiency impairs iTreg development. A. Foxp3 expression after 3-day (left) or 7-day (right) culture of naive CD4^+^ T cells stimulated with plate-bound anti-CD3 and anti-CD28 in the presence of 100 U/ml rhIL-2 and 10 ng/ml rhTGF-β_1_. n = 6–14 individual experiments per condition. *** P<0.001, Tukey's multiple comparison test. n.s.  = not significant. Day 3 and day 7 data are not from the same experiments. B. Median fluorescence of Foxp3 staining on CD4^+^Foxp3^+^ T cells after 7-day culture as in A. Matching symbols in either column in each plot correspond to paired samples. n = 6–9. No significant differences; paired, 2-tailed t-test. C + D. Mean expression (C) and representative cytometry plots (D) of transcription factors and surface markers on CD4^+^Foxp3^+^ T cells after 7-day culture as in A. n = 3–12 individual experiments per condition. C * P<0.05, ** P<0.01, *** P<0.001, one-way ANOVA with Dunnett's post-test.

iTreg cells were generated from naive CD4^+^ splenic T cells of wild-type Tg4, Tg4 IL-10^−/−^ or Tg4 CTLA-4^−/−^ mice in order to assess the role of these two mediators of immune suppression in their differentiation. As depicted in Figure 3A, deficiency in CTLA-4 but not IL-10 impaired anti-CD3 + anti-CD28-mediated Foxp3 induction. The effect of CTLA-4 deficiency on the frequency of Foxp3-expressing cells in the culture was clear from an early stage of differentiation at day 3 of culture (Foxp3 mean ± SEM of 76.6%±1.5 for wild-type Tg4, 77.4%±1.6 for Tg4 IL-10^−/−^ and 27.6%±6.7 for Tg4 CTLA-4^−/−^) and remained, albeit less pronounced, until day 7 (95.0%±0.9 wild-type, 95.2%±0.7 Tg4 IL10^−/−^, 77.0%±5.2 Tg4 CTLA-4^−/−^). This effect of CTLA-4 was demonstrated previously [5], yet contradicted by a recent study [6]. The activation status of the starting CD4^+^ T cell population is of pivotal importance for Foxp3 induction and has been argued to explain the differences between these two studies. It cannot be excluded fully that differences in the starting populations rather than a direct effect of CTLA-4 in our system underlie the effect observed, but the use of highly purified naive CD4^+^ T cells (>96% CD62L^hi^CD44^lo^ in all cases) from mice aged only 4–7 weeks, in addition to the fact that Tg4 IL-10^−/−^ but not Tg4 CTLA-4^–/–^ mice develop EAE spontaneously at a very young age, would argue against pre-activation as an explanation for our results. Moreover, we demonstrated recently that addition of a blocking anti-CTLA-4 antibody during iTreg cell differentiation of naive Tg4 CD4^+^ T cells in response to specific antigen led to a small reduction in frequency of Foxp3^+^ cells [17], thus supporting the findings reported here.

Although the percentage of cells expressing Foxp3 was significantly lower in Tg4 CTLA-4^−/−^ T cells, the level of Foxp3 expression per cell was not affected (Figure 3B). In addition to impaired Foxp3 induction, Tg4 CTLA-4^−/−^ cells, but not Tg4 IL-10^−/−^ cells, demonstrated an altered expression pattern of molecules commonly associated with Treg cell function, including Neuropilin-1, CD103 and CD62L (Figure 3C and 3D).

### iTreg cells require IL-10 for suppression of EAE

**Figure 4:**
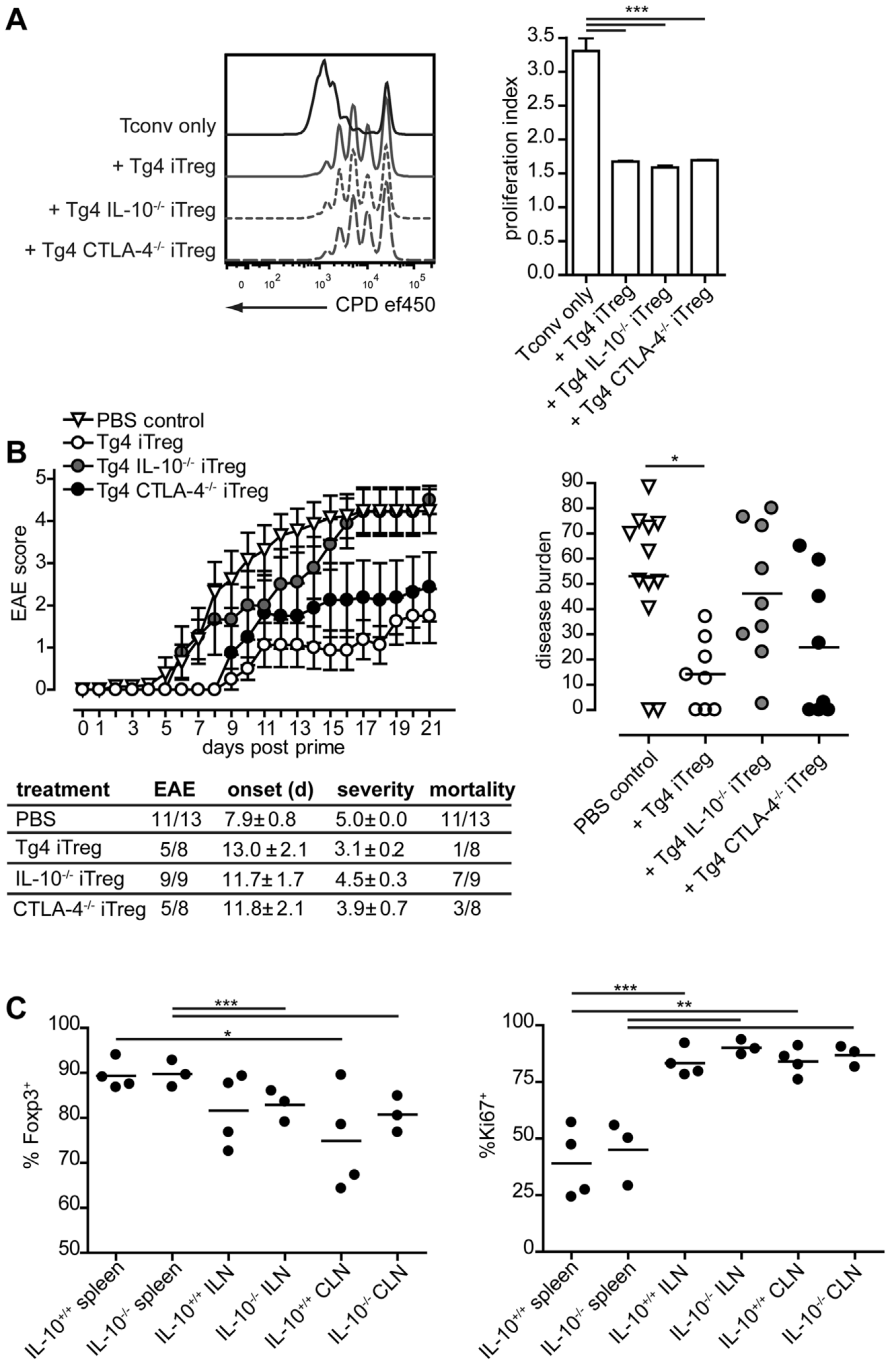
iTreg cell function is mediated by IL-10 and CTLA-4. A. CPD-ef450-labeled naive CD4^+^ Tg4 CD45.1^+^ T cells cultured in vitro for 4 days with or without Tg4 CD45.2^+^ iTreg cells and stimulated with 1 µg/ml MBP Ac1–9 and irradiated APCs. Representative overlays gated on CD45.1^+^ cells (left) and proliferation index as means of triplicates + SEM (right). Representative of 3 or more similar experiments. *** P<0.001, one-way ANOVA with Dunnett's post-test. B. Tg4 Rag^−/−^ mice received 5×10^6^ iTreg cells in PBS or PBS only, i.p., 3 days prior to EAE induction. Data in each line were combined from 2 or more individual experiments and are shown as mean ± SEM. Distribution plot shows disease burden for each individual in each group * P<0.05, Kruskal Wallis with Dunn's post-test comparing all pairs of columns. No statistically significant differences exist between other column pairs. C. Tg4 CD45.1^+^ mice received 5×10^6^ Tg4 CD45.2^+^ iTreg cells, i.p., 72 hrs prior to priming with MBP Ac1–9 in CFA. On day 7 post-prime, spleens, inguinal lymph nodes (ILN) and cervical lymph nodes (CLN) were removed and isolated cells analyzed for Foxp3 (left) and Ki67 (right) expression by FACS. Accumulated results from 4 Tg4 IL-10^+/+^ and 3 Tg4 IL-10^−/−^ mice, gated on CD45.2^+^ cells. ** P<0.01, *** P<0.001, Tukey's multiple comparison test.

The diverse effects of CTLA-4 and IL-10 on iTreg cell differentiation suggested a possible divergent effect on iTreg cell function. First, this was examined in vitro. CD4^+^CD62L^+^ Tconv cells from Tg4 CD45.1^+^ mice, labeled with CPD-ef450 were cultured at a 1 to 1 ratio with iTreg cells generated from wild-type Tg4, Tg4 IL-10^−/−^ or Tg4 CTLA-4^−/−^ mice (all CD45.2^+^) for 4 days and stimulated with 1 µg/ml MBP Ac1–9 and irradiated APC. In this in vitro setting, all varieties of iTreg cells suppressed the proliferation of Tconv cells equally (Figure 4A). This, however, was not the case in vivo. 5×10^6^ iTreg cells were transferred to sex-matched Tg4 Rag^−/−^ mice, 3 days prior to priming for EAE. In these recipient mice, which lack endogenous development of Foxp3^+^ Treg cells and therefore rely solely on cells transferred for immune regulation, wild-type iTreg cells significantly impaired disease development in terms of total disease burden over the duration of the experiment (Figure 4B). Furthermore, adoptive transfer of wild-type iTreg cells reduced the disease incidence, delayed disease onset, and lessened the mean severity in cases of EAE and, consequently, the number of cases that required early termination. This protective effect was impaired with Tg4 IL-10^−/−^ iTreg cells, increasing not only the disease burden compared to wild-type iTreg cells but notably also the disease incidence, severity and mortality. Recipients of Tg4 CTLA-4^−/−^ iTreg cells also showed a greater incidence of cases with high disease burden than recipients of wild-type iTreg cells but this may be due to the lower frequency of Foxp3 expression in the Tg4 CTLA-4^−/−^ iTreg cells transferred compared to wild-type iTreg cells (85% vs 95%) rather than the inability to express CTLA-4. This may have led to the transfer of a higher number of potential self-reactive effector T cells in addition to a somewhat lower number of Foxp3^+^ cells, which may have augmented disease severity. Foxp3 expression in transferred Tg4 IL-10^−/−^ iTreg cells was comparable to wild-type iTreg cells, with the deficiency in the ability to produce IL-10 the only obvious phenotypic difference. These results are in line with the reported role of IL-10 in iTreg cell-mediated amelioration of murine colitis [8]. Moreover, these results confirm previous findings using polyclonal iTreg cells in the control of MOG-induced EAE, indicating that antigen-specificity does not affect the need for IL-10 as the suppressive agent [7].

Finally, based on literature published, the reduced functionality of Tg4 IL-10^−/−^ iTreg cells most likely results from the mechanism reported previously through which iTreg cell-derived IL-10 suppresses the expression of co-stimulatory molecules by dendritic cells [6]. An alternative explanation is that IL-10-deficiency may impair the stability of the iTreg cell phenotype after disease induction. In order to assess this theory, Tg4 IL-10^+/+^ or Tg4 IL-10^−/−^ iTreg cells (both CD45.2^+^ and equal in Foxp3 expression) were transferred into sex-matched Tg4 CD45.1^+^ recipients 3 days prior to disease induction. On day 7 post-prime, spleens, inguinal lymph nodes (ILN) and cervical lymph nodes (CLN) were removed and the transferred iTreg cells in these organs were examined for remaining Foxp3 expression as well as the marker of cell division, Ki67. As shown in figure 4C, there was no difference in Foxp3 retention between Tg4 IL-10^+/+^ and Tg4 IL-10^−/−^ iTreg cells in any of these three lymphoid organs. However, iTreg cells in the lymph nodes had lost more Foxp3 expression than iTreg cells in the spleen, which correlated with greater Ki67 staining and thus proliferation. This finding of activation-driven instability is in line with a recent report [18]. It is clear from these results that the impaired functionality of IL-10-deficient iTreg cells results from absence of the effect of IL-10 on other cells types and not on the stability of the regulatory phenotype.

Overall, we demonstrate that adoptive transfer of antigen-specific iTreg cells can ameliorate development of MBP-mediated EAE. This prophylactic benefit of iTreg cell transfer is promising for future clinical use, although this may require further demonstration of efficacy after disease onset. The finding that CTLA-4 deficiency impairs the differentiation of iTreg cells in our model is of particular interest. After all, in the same self-antigen-specific mouse model we previously found that CTLA-4 tunes the thymic development of Tconv and Treg cells [1], [4], leading to a greater induction of Foxp3 expression in Tg4 CTLA-4^−/−^ mice. Although in vitro differentiated iTreg cells may not be directly comparable to naturally occurring extra-thymic Foxp3^+^ pTreg cells and caution is required when interpreting these in vitro findings, they could suggest that, in response to the same thymically expressed self antigen, CTLA-4 differentially regulates thymic and extra-thymic induction of Foxp3.

Our findings confirm that IL-10 is essential for iTreg cell-mediated immune regulation, thus demonstrating that this cytokine is pivotal for the function of both Foxp3^−^ and Foxp3^+^ in vitro differentiated Treg cell subtypes and therefore of fundamental importance across the scope of immunotherapy based on Treg cell transfer. Despite the use of this common mediator of immune regulation, there are great differences in the generation and function of the different extra-thymic Treg cells subsets; further experiments are required to reveal whether treatment of MS or any other autoimmune disease would benefit more from the transfer or in vivo induction of IL-10-secreting Foxp3^−^ T cells or the adoptive transfer of Foxp3^+^ iTreg cells in order to restore a healthy immune balance. Finally, ongoing work will have to demonstrate if our findings presented here on in vitro-differentiated iTreg cells carry relevance to Foxp3^+^ pTreg cells generated therapeutically in situ.

## Acknowledgments

The authors wish to thank Mrs. Louise Falk, Miss Anna Lewis and the UoB ASU for assistance with animal breeding and maintenance and Prof. Michael Day for histological examination. Also, we thank Dr. Andrew Herman at the UoB FMVS Flow Cytometry Facility for advice and assistance.
